# Surgical dose and the clinical outcome in the treatment of mammary gland tumours in female dogs: a literature review

**DOI:** 10.1186/s13028-023-00674-1

**Published:** 2023-03-11

**Authors:** Maria Bennet Hörnfeldt, Jacob Kvesel Mortensen

**Affiliations:** Gothenburg Animal Hospital Evidensia/Göteborgs Djursjukhus Evidensia, Produktvägen 5, 435 33 Mölnlycke, Sweden

**Keywords:** Canine, Lumpectomy, Mastectomy, Outcome

## Abstract

Mammary gland tumours are the most frequent tumours in intact female dogs and surgery remains the main treatment modality. Surgery is traditionally performed according to the lymphatic drainage of the mammary glands, but robust evidence is still lacking on what surgical dose is the smallest and results in the best outcome. The objective of the study was to investigate whether choice of surgical dose influences treatment outcome in dogs with mammary tumours and to identify current gaps in research that need to be filled in future studies for identifying the smallest surgical dose with the best possible outcome. Articles for entrance into the study were identified in online databases. Information regarding outcome following use of different surgical doses was extracted for analysis. Also, known prognostic factors were mapped for each study to discuss their impact on treatment outcome. Twelve articles were identified and included. Surgical dose applied ranged from lumpectomy to radical mastectomy. Radical mastectomy was most often analysed [11/12 (92%) articles]. Less invasive surgical doses were used less often in decreasing order of invasiveness. Outcomes analysed were most often survival time [7/12 (58%) articles], frequency of recurrences [5/12 (50%) studies] and time to recurrence [5/12 (42%) studies)]. No studies demonstrated any significant association between surgical dose and outcome. Gaps in the research could be categorised as data that was not available for extraction, for example known prognostic factors. Other factors related to study design were also identified, for example small groups of dogs included into the study. No studies showed a clear benefit of choosing one surgical dose over the other. Choice of surgical dose should be based on known prognostic factors and risks for complications rather than on lymphatic drainage. In future studies all prognostic factors should be included when investigating how choice of surgical dose influences treatment outcome.

**Supplementary Information:**

The online version contains supplementary material available at 10.1186/s13028-023-00674-1.

## Background

Mammary gland tumours are common in dogs, and they are the most common neoplasms in sexually intact female dogs [1–3]. The incidence is higher in many European countries when compared to the United States where female dogs are more commonly ovariohysterectomised at an early age [2, 4, 5].

Tumour size, ulceration, fixation to underlying structures, lymph node status and stage are strong predictive factors of prognosis. Dogs with tumour diameter larger than 3 cm have a statistically significant worse outcome compared to dogs with smaller tumours [6–8]. This has also been supported by the finding of histological progression from benign to malignant with increasing tumour size [6]. Ulceration has been found to be an independent predictor of poor prognosis [9] and fixation to underlying structures has shown to significantly shorten the duration of the metastasis-free interval [10]. Also, the completeness of the surgical margins is also a strong prognostic factor as clean margins result in a better outcome [9, 11].

The lymphatic system represents the main route of metastasis for mammary cancer in dogs [12–14]. The lymphatic route has therefore traditionally aimed in clinical decision making when choosing the appropriate surgical dose for mammary tumours in dogs. However, performing mastectomy based on the lymphatic route has been questioned because of the tumour’s ability to alter the lymphatic drainage by creating ipsilateral and contralateral anastomoses [15]. Therefore, particularly in dogs with malignant mammary tumours, it is unclear whether choice of surgical dose based on lymphatic drainage influences treatment outcome [16].

In human medicine there has been a shift in treatment practice for women with breast cancer throughout the last 30 years as 60–80% of newly diagnosed mammary cancer cases are amenable to wide local excision [17]. In some women with mammary cancer, mastectomy is still carried out because of large tumour sizes, tumour multicentricity or inability to achieve clean surgical margins after multiple resections. In dogs, there is still a need to investigate how to choose the simplest procedure that will result in removal of all neoplastic tissue [16]. Also, the risk of intraoperative and postoperative complications is higher when performing radical mastectomy compared to regional mastectomy and should therefore be taken into consideration [18]. Smaller mammary gland tumours less than 3 cm in size are, however, very common and comprise up to 55% of mammary tumours at presentation [8, 19, 20]. This high frequency could indicate that many owners are quick to seek veterinary advice if they identify tumours in the mammary gland. This could lead to treatment of mammary tumours at the earliest stage possible and optimal long-term outcome with the smallest surgical dose.

In this scoping review eligible studies for inclusion focused on the efficacy of surgical doses of various extent in the treatment of female dogs with mammary neoplasia and identification of gaps in the research. The scope of the enquiry was therefore guided by the following research question:

In female dogs with mammary neoplasia, does surgical dose influence treatment outcome?

## Search strategy

The present review is a scoping review [21]. This structure of a review was chosen to present a broader picture of the literature available on the subject and to identify current research gaps that would need to be filled to make future clinical guidelines on how to choose the simplest procedure resulting in the best possible outcome in treatment of canine mammary neoplasia.

We considered studies that included female dogs of all ages that received surgical treatment for malignant mammary neoplasia and had the diagnosis confirmed by histopathology. There was no age limitation because mammary neoplasia can occur in dogs of most ages even though it is most common in middle-aged and older dogs.

Studies considered eligible for inclusion into this study were randomized controlled trials, prospective cohort studies, case–control studies and case series (prospective or retrospective). Case reports and expert opinions were not considered eligible. Studies were excluded if it was not possible to retrieve a full-text article.

Studies were included only if they analysed dogs with malignant mammary neoplasia treated surgically, but studies including dogs with both malignant and benign mammary neoplasia were also included as it was expected that some studies would include dogs with tumours of both biological behaviours.

Studies were included if they provided a comparison of the outcome in dogs with mammary neoplasia treated with different surgical doses or if they reported on the outcome for dogs treated with a single surgical dose without including a control group. Studies were included only if they had follow-up on included dogs for a minimum of 1 year after surgery. Studies on inflammatory carcinoma were excluded. Studies on dogs given adjuvant medical treatment were excluded except in cases where medical treatment showed no effect on outcome. The comparators were categorised according to the extent of surgical dose including lumpectomy, simple mastectomy, regional mastectomy and radical mastectomy.

The surgical doses were defined as follows [22]:

Lumpectomy: removal of the tumour only.

Simple mastectomy: removal of the affected gland only.

Regional mastectomy: removal of the affected gland and glands that shared lymphatic drainage along with removal of associated lymph nodes.

Radical mastectomy: removal of the entire mammary chain and associated lymph nodes either unilaterally or bilaterally.

The context of the present review was studies reporting on the efficacy of surgical doses of various extent for treatment of mammary tumours in female dogs in Europe and North America. Studies from other continents were also screened and included whenever they were found relevant for the research question. Studies published in English and German were considered for inclusion in the review. No limitation on publication date was imposed upon the literature search.

Outcome measures in the studies included into this review were grouped into the following definitions:

- Time to recurrence: time interval between the day of reference in the study (e.g., date of diagnosis or treatment) and the day of recurrence (local recurrence, regional metastases or distant metastases)
- Frequency of recurrences: the rate of recurrences (local recurrence, regional metastases or distant metastases) at the end of the follow-up period
- New mammary lesion development frequency: the number of lesions developed in the remaining mammary tissue during the follow-up period
- Mortality rate: the rate of deaths within the follow-up period
- Survival time: time interval between the day of reference in the study (e.g., date of diagnosis) and the day of death

Along the extraction of outcome variables, information about some pre- and postoperative parameters were also extracted for discussion of their value in analysis of the outcome. These parameters included: prior treatment (surgery/chemotherapy/radiation), number of tumours per dog, largest tumour size, percentage of dogs with ulcerated tumours, percentage of dogs with tumours fixated to underlying tissue, percentage of dogs with stage I and percentage of dogs with clean surgical margins.

The search strategy of this scoping review followed a three-step search method as recommended for standard JBI systematic reviews [23].

The first step was an initial limited search on Ovid MEDLINE performed on 26th September 2019. Initial keywords used for this search were based on the keywords of the research question of the present study and synonyms and included:

- dog? OR canine OR canid?
- mammary
- tumour OR tumor OR neoplasia OR cancer OR lump
- surgery OR surgical OR mastectomy OR lumpectomy OR extirpation
- efficacy OR outcome

The five searches were combined with AND.

This initial search identified 34 articles as documented in Additional file 1. The search was followed by an analysis of keywords included in the title and abstract, and of the index terms used to describe the article. Key words identified are listed in Additional file 2.

The second step was a more comprehensive search using all identified keywords and index terms across all relevant databases including CAB Abstracts, Embase, BIOSIS Previews and MEDLINE. The title and abstract of articles identified in this step were screened for relevance. Articles that seemed relevant went on to full text screening and were either included or excluded based on the inclusion criteria and the articles’ relevance. The search results from the more comprehensive OVID database search are documented in Additional file 3.

As the third step, the reference lists of all identified articles were searched for additional studies.

Data was double extracted by the authors. Agreement on the extracted data to be included was reached by rereading the article with the data in question.

## Review

**Fig. 1:**
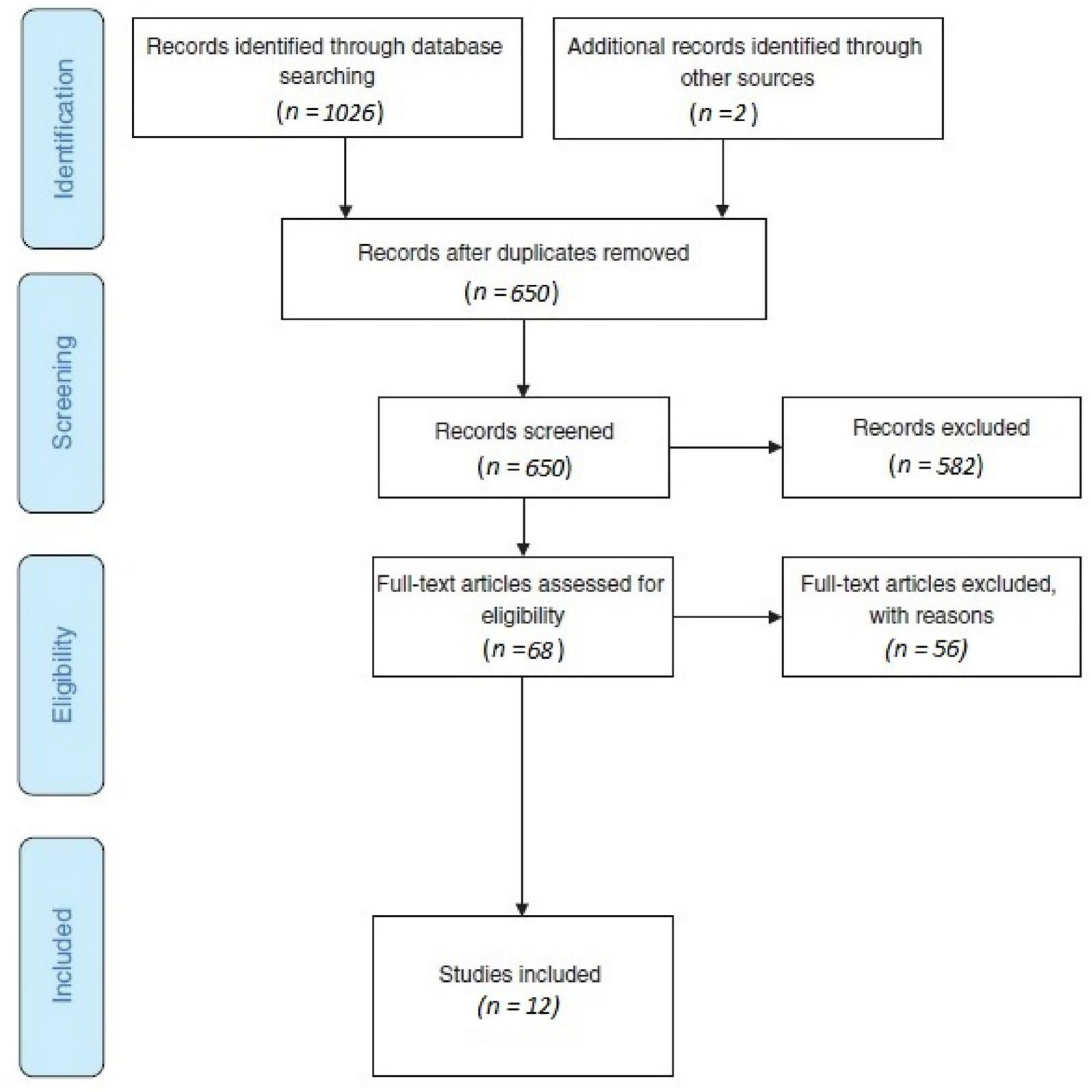
PRISMA flow diagram. The PRISMA flow diagram details the search and selection process applied in this literature review

The search strategy revealed a total of 1026 articles (PRISMA flow diagram, Fig. 1). Additionally, two articles were identified from reference list searching. After the removal of 378 duplicates and 582 articles irrelevant based on the title and abstract, full text of 68 articles were retrieved and read to determine relevance to the research question. Of these, 53 were excluded because they did not have enough information about the surgical method used and three were excluded because of adjuvant medical treatment. A total of 12 articles met the inclusion criteria. 10 studies were in English language and two were in German.

The studies included were published in the years 1976 to 2016 representing a 40-year period. Based on the country of the first author most studies, 9/12 (75%), were performed by authors located in Europe and USA. Also, studies from Brazil, Taiwan and Japan were included and comprised the remaining 3/12 (25%) studies. Most studies were performed on dogs presented at university hospitals, 9/12 (75%), and a subset of studies, 3/12 (25%), were performed on dogs presented at private practices.

The study type was most often of descriptive nature; hence 8/12 (67%) studies were case series and the remaining 4/12 (33%) were of analytical nature; randomised controlled trials or prospective cohort studies.

The number of dogs included into each study varied between 31 and 253 and most studies included 100–149 dogs (Table 1). Information about prior treatment was available in 5/12 (42%) studies of which prior surgical treatment was given to a subset of 19% of dogs in one study by Wey et al. [24]. In the study by Betz et al. [25] no prior chemotherapy or radiation was given to the patients, but it was not possible to determine whether any patients had prior surgery.

**Table 1:** Summary of extracted data from 12 studies

	Number of studies (%)
Number of dogs	
0–49	1 (8%)
50–99	4 (33%)
100–149	6 (50%)
150+	1 (8%)
Prior treatment	
Surgery	1 (8%)
No prior treatment	4 (33%)
Not available	7 (58%)
Number of tumours per dog	
1	1 (8%)
2+	5 (42%)
Not available	6 (50%)
Median/mean tumour size (in majority of dogs)	
< 3 cm	5 (42%)
≥ 3 cm	3 (25%)
Not available	4 (33%)
Percentage of dogs with ulcerated tumours	
< 50%	2 (17%)
Not available	10 (83%)
Percentage of dogs with tumours fixated to underlying tissue	
< 50%	2 (17%)
Not available	10 (83%)
Percentage of dogs with stage I	
< 50%	1 (8%)
≥ 50%	4 (33%)
Not available	7 (58%)

Information about the number of tumours per dog was provided in 6/12 (50%) studies. The study by Stratmann et al. [26] included only dogs with one tumour at presentation whereas in the remaining five studies most dogs had more than one tumour.

Information about tumour size was provided in 8/12 (67%) studies. In 5/8 (63%) studies the median or mean tumour size was less than 3 cm. In general, however, there was a large variation in tumour size ranging from 0.3 to 21.0 cm in diameter. The study by Stratmann et al. [26] included only dogs with tumours less than 3 cm whereas the other studies included tumours of all sizes. The percentage of dogs with ulcerated tumours was available in 2/12 (17%) studies and ranged from 8 to 18%. Also, percentage of dogs with tumours fixated to underlying tissue was available in 2/12 (17%) studies and ranged from 8 to 18%. In Allen et al. [27] invasion into skin, muscle or body wall was incorporated into an older classification system and it was therefore not possible to separate dogs with ulcerated tumours from dogs with tumours fixated to underlying tissue. Even though the percentage of dogs with ulcerated and fixated tumours could only be extracted in 2/12 studies the influence of these two parameters on outcome was still analysed in a total of 7/12 (58%) studies [11, 25, 26, 28, 30–32]. Stage of the mammary tumours was available in 5/12 (42%) studies. Most often dogs had stage I even though there was a large variation in percentage of dogs with stage I ranging from 6 to 100%.

The surgical dose used ranged from lumpectomy to radical mastectomy, unilateral or bilateral. The least extensive surgical dose investigated was most often simple mastectomy or regional mastectomy comprising 8/12 (67%) studies. In contrast most studies, 11/12 (92%), included dogs treated with radical mastectomy for comparison, either unilateral or bilateral. Groups of dogs treated only with lumpectomy most often included smaller numbers of dogs when compared to groups of dogs treated with more invasive doses.

The biological behaviour of the tumours was malignant only in 6/12 (50%) studies. In the remaining studies dogs with both malignant and benign tumours were included, most often malignant except in Betz et al. [25] and Itoh et al. [33] where most dogs had benign tumours. Surgical margin status was known in only 2/12 (17%) studies where all margins were clean in Stratmann et al. [26] and 49% were clean in Misdorp et al. [28, 29]. Follow-up time for the studies varied from 1 to > 5 years.

Outcome was measured in multiple different ways in the studies included, most often survival time, 7/12 (58%) studies, frequency of recurrences, 5/12 (50%) studies, and time to recurrence, 5/12 (42%) studies. Surgical dose had no influence on outcome in any of the included studies. In Stratmann et al. [26] all dogs had one single T1NxM0 tumour and were treated with regional mastectomy. As 58% of dogs in this study developed a new tumour in the ipsilateral side, the authors recommended radical mastectomy rather than regional mastectomy. The results of all articles included are summarised in Table 2.

**Table 2:** Summary of each article included

Author and year	Country/study design/setting/aim of the study	Prior treatment/tumour characteristics/number of dogs and treatment/follow up time	Outcome/key findings
MacEwen 1985 [7]	USA Randomized controlled trial University setting Aim: to evaluate the effect of levamisole and surgery on canine mammary cancer	No prior treatment **Simple mastectomy: 72 dogs** **Radical mastectomy: 72 dogs** All dogs had malignant tumours All dogs were followed up every 2 months until death, but not all dogs had died when the study was published	Outcomes: TTR and survival time MST not reached for dogs treated with simple mastectomy **Surgical technique had no influence on outcome**
Simon 2006 [10]	Germany Randomized controlled trial University setting Aim: to investigate whether adjuvant doxorubicin or docetaxel will improve the treatment outcome in dogs with high-risk malignant mammary gland tumours and whether the use of docetaxel will be feasible in affected dogs	No prior treatment Number of tumours per dog: median 3 (range, 1–9) Diameter of largest tumour: median 6.6 cm (range, 1.4 – 11.5 cm) Stage I (T1N0M0): 6% of dogs (n = 31) **Regional mastectomy: 17 dogs** **Radical mastectomy: 14 dogs** All dogs had malignant tumours Follow-up time: 4 years (median 258 days, range 13–2585 days)	Outcomes: TTR (local or distant metastases) and MST Median not reached for recurrence-free interval **Surgical technique had no influence on outcome**
Pena 2012 [30]	Spain Prospective cohort study University setting Aim: to describe and evaluate a canine-adapted histological grading method of canine mammary tumours as a prognostic indicator in a prospective study	Number of tumours per dog: median 1 (range, 1–3) Diameter of largest tumour: mean 2.2 cm (range, 0.5–14.0 cm) Stage I: 65% of dogs (n = 65) **Lumpectomy, simple, regional or radical mastectomy: 65 dogs** All dogs had malignant tumours Follow-up: 28–38 months	Outcomes: frequency of recurrences, TTR, mortality rate and survival time **Surgical technique had no influence on outcome**
Betz 2012 [25]	Germany Prospective cohort study University setting Aim: to characterize outcome following surgery and identify independent prognostic factors in canine mammary tumours	No prior treatment with chemo or radiation Number of tumours per dog: median 2 (range, 1–9) Diameter of largest tumour: median 2.5 cm (range, 0.3–14.0 cm) Stage I: 57% of dogs (n = 134) **Simple mastectomy: 30 dogs** **Regional mastectomy: 41 dogs** **Radical mastectomy: 63 dogs** Malignant tumours: 24% of dogs (n = 134) Follow-up: 4 years	Outcomes: frequency of recurrences (local), TTR (local recurrence and distant metastases), survival time **Surgical technique had no influence on outcome**
Stratmann 2008 [26]	Germany Prospective case series University setting Aim: to investigate the histologic diagnosis and incidence of new mammary tumour growth in the remaining mammary chain tissue after regional mastectomy	No prior treatment Number of tumours per dog: 1 Diameter of largest tumour: mean 2.2 cm (range, 1–3 cm) All dogs had stage T1NxM0 **Regional mastectomy: 99 dogs** Malignant tumours: 74% of dogs (n = 99) All tumours had clean margins Follow-up: median 3.8 years (range, 1–5 years)	Outcomes: new mammary lesion development frequency and TTR New mammary lesion development frequency: 58% of dogs (n = 99) (these dogs developed a new tumour in the ipsilateral chain) TTR, range 1–60 months **The authors recommended radical mastectomy rather than regional mastectomy because of the high frequency of tumour recurrence in ipsilateral chain**
Misdorp 1976 [31]	Netherlands Retrospective case series Private practice setting Aim: to analyse 10 mammary tumour characteristics in dogs with mammary cancer with special reference to their association with prognosis	**Simple mastectomy: 59 dogs** **Radical mastectomy: 42 dogs** All dogs had malignant tumours Follow-up: 2 years	Outcome: mortality rate Surgical technique had no influence on outcome. **Surgical technique had no influence on overall outcome, but simple mastectomy gave better outcome in dogs with noninvasive tumours less than 5 cm and not involving surrounding tissue and radical mastectomy gave better outcome in dogs with severely infiltrating tumours**
Misdorp 1979 [28, 29]	Netherlands Retrospective case series Private practice setting Aim: to analyse 14 tumour and host characteristics for association with prognosis in dogs surgically treated for mammary cancer	**Simple mastectomy: 211 dogs** **Radical mastectomy: 42 dogs** All dogs had malignant tumours Clean margins: 49% of cases (n = 178) Follow-up: 2 years	Outcomes: frequency of recurrences (local or distant metastases), survival time **Surgical technique had no influence on overall outcome, but in dogs with smaller low-grade tumours simple mastectomy gave better outcome. In dogs with high-grade tumours radical mastectomy gave better outcome**
Allen 1989 [27]	USA Retrospective case series University setting Aim: to evaluate prognostic value of specific physical findings, histological type and relative effects of different types of surgical excision in dogs with mammary cancer	63% of dogs (n = 128) had more than one tumour Invasion into skin, muscle or body wall: 18% of dogs (n = 97) (not possible to separate ulcerated tumours from tumours fixated to underlying tissue) **Lumpectomy: 18 dogs** **Simple mastectomy: 6 dogs** **Regional mastectomy: 13 dogs** **Radical mastectomy (unilateral): 14 dogs** **Radical mastectomy (bilateral): 18 dogs** Malignant tumours: 65% of dogs (n = 97) Follow-up: > 1 year	Outcome: Frequency of recurrences (local) **Surgical technique had no influence on outcome** A surgical margin of 2 cm or more from the lesion is suggested as enough to minimize patient morbidity
Wey 1999 [24]	Germany Retrospective case series University setting Aim: to evaluate incidence, age and breed of dogs with mammary tumours as well as prognosis following surgical treatment	Prior surgery: 19% of dogs (n = 75) Number of tumours per dog: mean 6.9 Ulceration: 8% dogs Fixation to underlying tissue: 8% dogs Diameter of largest tumour: < 3 cm in 64% of dogs (stage I) (n = 75) **Lumpectomy: 1 dog** **Regional mastectomy: 15 dogs** **Radical mastectomy: 59 dogs** Malignant tumours: 83% of dogs (n = 75) Follow-up: 1.5–2.5 years	Outcome: frequency of recurrences (local recurrences, regional and distant metastases) **Surgical technique had no influence on outcome**
Itoh 2004 [33]	Japan Retrospective case series Private practice setting Aim: to evaluate clinical outcomes of both benign and malignant mammary gland tumours with concern to the differences between small-breed dogs and others	Diameter of largest tumour: < 3 cm in 67% of dogs (n = 81) **Regional or radical mastectomy (unilateral or bilateral): 101 dogs** Malignant tumours: 39% of dogs (n = 101) Follow-up: > 1 year	Outcome: mortality rate **Surgical technique had no influence on outcome for carcinoma cases**
Chang 2005 [22]	Taiwan Retrospective case series University setting Aim: to identify prognostic factors for female dogs that have undergone surgical removal of malignant mammary tumours	Diameter of largest tumour: mean ± SD 7.2 ± 4.9 cm (range, 0.5–21 cm) **Lumpectomy: 7 dogs** **Simple mastectomy: 24 dogs** **Regional mastectomy: 33 dogs** **Radical mastectomy: 10 dogs** All dogs had malignant tumours Follow-up: 2 years	Outcome: survival time **Surgical technique had no influence on outcome**
Dias et al. [32]	Brasil Retrospective case series University setting Aim: to investigate the relationship between survival time after mastectomy and a number of clinical and morphological variables	Diameter of largest tumour: < 3 cm in 49% of dogs (n = 143) **Regional mastectomy, unilateral radical mastectomy (38% of dogs, most frequent) or bilateral mastectomy: 139 dogs** Malignant tumours: 77% of dogs (n = 143) Follow-up: up to 64 months	Outcome: survival time **Surgical technique had no influence on outcome**

Main findings relevant for the objective of this study are highlighted in bold

Gaps in the research could be categorised as information that was not available for analysis based on the extraction tables designed for the present study. Surprisingly, the most frequently missing information was the status of surgical margins, i.e., whether there were clean margins on the tumours removed and comprised 10/12 (83%) studies. Other missing information was whether any prior treatment was given [7/12 (58%) studies], number of dogs with stage I [7/12 (58%) studies], number of tumours per dog [6/12 (50%) studies] and largest tumour diameter [4/12 (33%) studies]. Gaps in the research were also identified by subjective comparison of the studies included, such as small numbers of dogs in each treatment group or older staging systems being used. Identified gaps in research for each article are summarised in Table 3.

**Table 3:** Identified gaps in research for each article

	Prior treatment unknown	Number of tumours per dog unknown	Largest tumor diameter unknown	Number of dogs with ulcerated tumours unknown	Number of dogs with tumours fixated to underlying tissue unknown	Stage unknown	Surgical margin status unknown	Other
MacEwen et al. [7]		x	x	x	x	x	x	
Simon et al. [10]				x	x		x	Some other known prognostic factors (e.g. tumour size and stage) lost their impact on treatment outcome because of small number of patients Low number of dogs with stage I
Pena et al. [30]	x			x	x		x	No information on how type of surgery was selected for each case
Betz et al. [25]				x	x		x	Type of surgery was chosen based on surgeon’s preference (no standardization) Uneven distribution of benign and malignant tumours when compared to other studies Histopathology on local recurrences and metastases was only performed on a limited number of cases
Stratmann et al. [26]				x	x			No control group Incomplete staging (status of local lymph node unknown) No subgrouping according to tumour size (1, 2 and 3 cm tumours)
Misdorp and Hart [31]	x	x	x	x	x	x	x	Old staging system
Misdorp and Hart [28, 29]	x	x	x	x	x	x		Old staging system High percentage of dirty margins
Allen and Mahaffey [27]	x		x			x	x	Low numbers of patients in each treatment group Old staging system Invasion into skin, muscle or body wall was incorporated into an older classification system and it was therefore not possible to separate dogs with ulcerated tumours from dogs with tumours fixated to underlying tissue
Wey et al. [24]							x	
Itoh et al. [33]	x	x		x	x	x	x	Low number of malignant tumours Low number of dogs eligible for survival analysis (14 dogs)
Chang et al. [22]	x	x		x	x	x	x	Small groups of dogs Stages I, II and III were grouped so stage I cases could not be extracted
Dias et al. [32]	x	x		x	x	x	x	Unknow number of dogs within each treatment group

## Discussion

The purpose of this scoping review was to investigate whether surgical dose influences treatment outcome in female dogs with mammary neoplasia. Only a minority of studies included (2/12) were randomised controlled trials. Despite representing the highest level of evidence, they can, however, be vulnerable to some weaknesses. For example, in Simon et al. [10] the number of dogs included in each group was very low when compared to the study by MacEwen et al. [7]. This meant that some other known prognostic factors (e.g., tumour size and stage) lost their impact on treatment outcome in Simon et al. [10]. Also, treatment outcome might be different in studies with small groups of dogs when compared to large groups of dogs because the statistical power was insufficient to prove it. Other weaknesses in the two studies were differences in data that was not available for extraction. In MacEwen et al. [7] information about number of tumours and tumour size was not directly available and the staging system used was different from the TNM system that is currently used and based on the staging system published by Owens [34].

Prospective cohort studies comprised 2/12 of the studies included [25, 30]. Both studies were very diverse in tumour characteristics as there was a large variation in number of tumours per dog, tumour size and stage. Also, different surgical doses were used, 3–4 different doses per study. A limitation in the studies is the lack of information on how the surgical dose was chosen. In the case where more than one veterinarian performed the surgeries there is a risk of bias as preferences for surgical dose may vary between veterinarians.

Most studies included, 8/12, were case series, either prospective or retrospective. The prospective study by Stratmann et al. [26] was the only study that did not include a control group as all dogs were treated with regional mastectomy only. The study had several qualities considering its design. There was a relatively high number (99) of dogs included, and the dogs were very uniform in terms of tumour characteristics as all dogs had only one tumour and all tumours were below 3 cm in size and were stage T1NxM0. As stage is known to have prognostic value [6, 8] it makes sense to look at this group of dogs separately. Also, all surgical margins were clean on histopathology and follow-up time was long (median 3.8 years). Most dogs (58%) developed a new tumour in the remaining mammary glands, but it is not known whether a more radical surgery would have prevented new tumour growth or not and whether the development of the second tumour was associated with the first tumour or not. Among some of the case series there was a big variation in known clinical prognostic factors. For example, there was a big variation in tumour size and so it was not possible to compare the outcome of different surgical doses in groups of tumours of certain sizes. This was seen in Chang et al. [22] where the tumour size ranged from 0.5 to 21 cm. Using the same surgical dose for tumours of different sizes could potentially lead to differences in surgical margin status depending on the size of the tumour and could therefore influence the outcome because surgical margin status is a known prognostic factor [9, 11]. In future studies the variation in margin size could be minimised by looking at groups of tumours of certain sizes, for example in groupings based on stage.

Seven out of eight case series had groups of dogs treated with two or more different surgical methods and comparison between groups was generally possible. These seven studies all included dogs treated with radical mastectomy and reflects that this has generally been a preferred surgical method. There might be several reasons to this finding, for example that many studies included many dogs with multiple tumours and that the size of the tumours was very different. Therefore, radical mastectomy might have been the only reasonable choice to obtain the best possible outcome considering the lymphatic drainage as route of metastasis and the surgeon’s preference and experience for choosing the surgical method. There were fewer dogs treated with lumpectomy, which could have been influenced by the surgeon’s preference when considering number of tumours, tumour size, stage and the ability to obtain clean margins. Also, the surgeon’s choice might have been influenced by treatment recommendations from human literature when breast cancer was more often treated with more radical surgery instead of less invasive surgery as recommended today [17].

All studies included groups of dogs treated with surgery based on the lymphatic drainage, which has traditionally aimed in the decision making when choosing surgical dose for treatment of mammary tumours [12–14] and therefore this finding was not a surprise. The lymphatic drainage might be modified in dogs with mammary tumours as Pereira et al*.* [15] showed an increased frequency of contralateral anastomoses between mammary glands in dogs with mammary tumours. However, the clinical relevance of the finding has not been confirmed, but it may explain why radical mastectomy was not superior to other less invasive surgical doses as the risk of contralateral anastomoses could increase the risk of new tumour growth and development of distant metastases.

## Conclusions

None of the studies included showed a clear benefit of choosing one surgical dose over another. The treatment most often used was radical mastectomy and the second most regional mastectomy. Dogs treated with simple mastectomy or less were less commonly represented and therefore it is not possible to conclude on what surgical dose is the least extensive that would result in the best possible outcome. As it was not possible to solely conclude on whether choice of surgical dose influences treatment outcome, the decision on what extent of surgery to perform should be based on known prognostic factors and the surgeon’s experience. Future studies should ideally be of analytical nature and include a control group, either randomised controlled trials or prospective cohort studies. Also, the inclusion of prognostic factors into multivariate analysis is important to find out whether any surgical method is an independent prognostic factor.

## Supplementary Information

**Additional file 1.** Initial OVID database search.

**Additional file 2.** Text words identified through searching the title and abstract and searching the index terms used to describe the articles.

**Additional file 3.** More comprehensive OVID database search.

## Data Availability

All data generated or analysed during this study are included in this published article. Do also see Additional files 1, 2 and 3.

## Abbreviations

MST: Median survival time
SD: Standard deviation
TTR: Time to recurrence
