# Supplementary figures and images for: Surgical dose and the clinical outcome in the treatment of mammary gland tumours in female dogs: a literature review
Source: Acta Vet Scand. 2023 Mar 11;65:12. doi: 10.1186/s13028-023-00674-1 (PMC10008593; doi:10.1186/s13028-023-00674-1)

Additional file 1. Initial OVID database search.


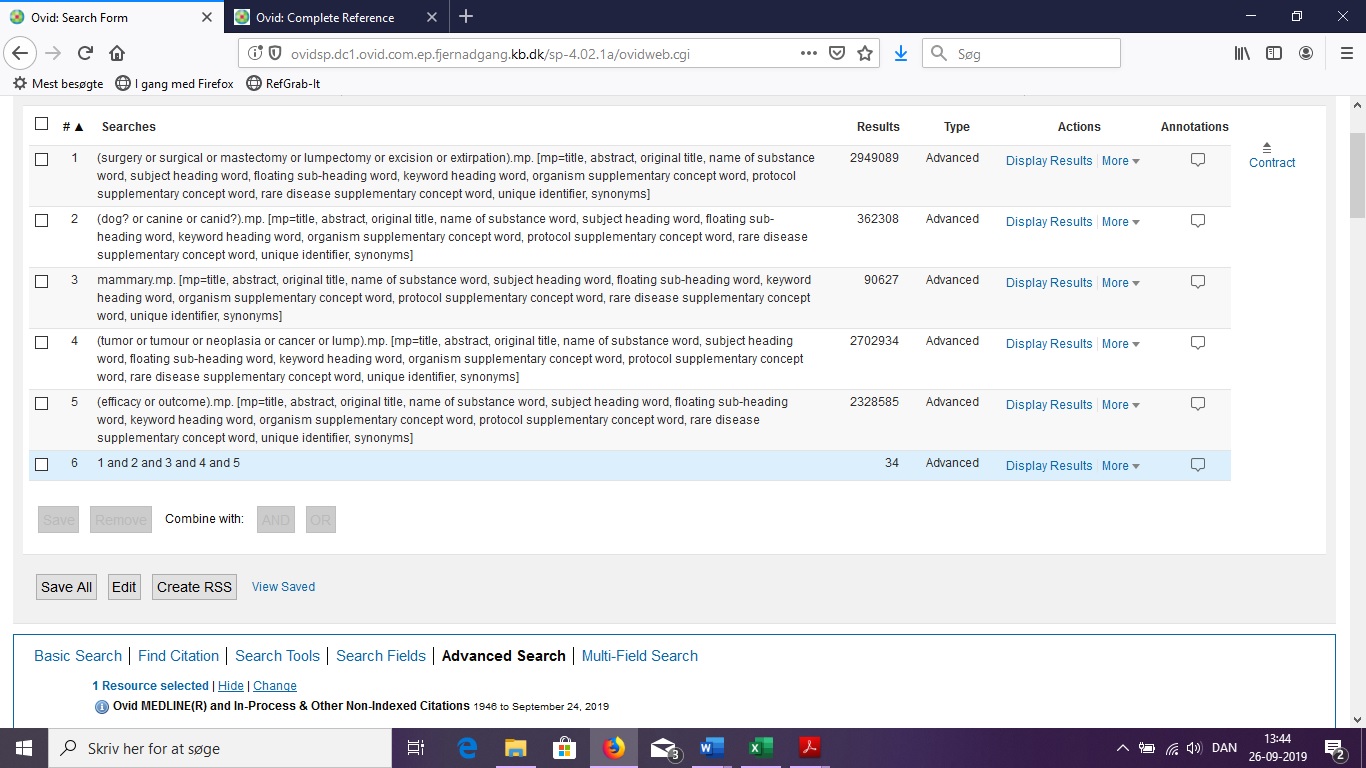

Supplement: Supplementary file 1 — Additional file 1. Initial OVID database search. [file 13028_2023_674_MOESM1_ESM.docx]

Additional file 3. More comprehensive OVID database search.


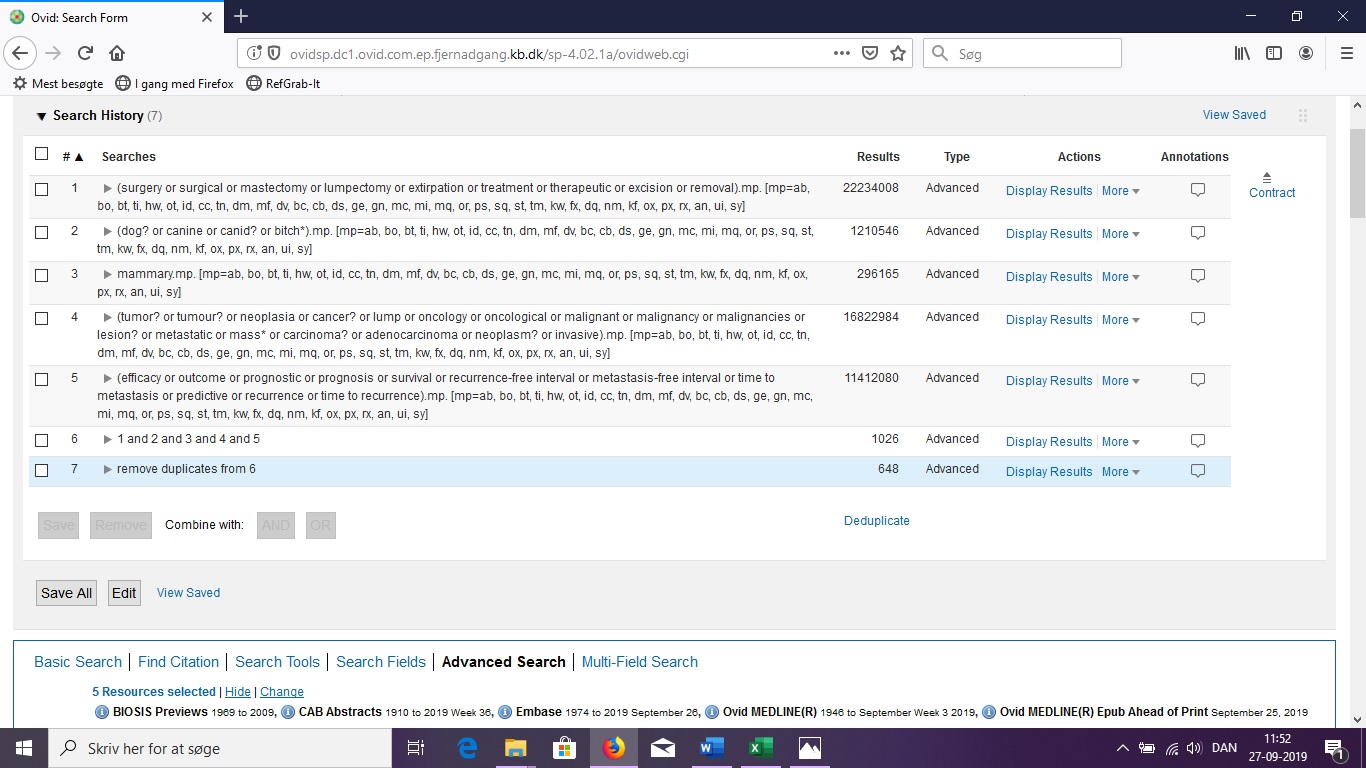

Supplement: Supplementary file 3 — Additional file 3. More comprehensive OVID database search. [file 13028_2023_674_MOESM3_ESM.docx]
