# Supplementary material for: Surgical dose and the clinical outcome in the treatment of mammary gland tumours in female dogs: a literature review
Source: Acta Vet Scand. 2023 Mar 11;65:12. doi: 10.1186/s13028-023-00674-1 (PMC10008593; doi:10.1186/s13028-023-00674-1)
Supplement: Supplementary file 2 — Additional file 2. Text words identified through searching the title and abstract and searching the index terms used to describe the articles. [file 13028_2023_674_MOESM2_ESM.docx]

Additional file 2. Text words identified through searching the title and abstract and searching the index terms used to describe the articles.

dog?

canine

canid?

bitch*

mammary

tumour?

tumour?

neoplasia

cancer?

lump

oncology

oncological

malignant

malignancy

malignancies

lesion?

metastatic

mass*

carcinoma?

adenocarcinoma?

neoplasm?

invasive

surgery

surgical

mastectomy

lumpectomy

extirpation

treatment

therapeutic

excision

removal

efficacy

outcome

prognostic

prognosis

survival

recurrence-free interval

metastasis-free interval

time to metastasis

predictive

recurrence

time to recurrence
